# Comparison the effect of charcoal-containing, hydrogen peroxide-containing, and abrasive whitening toothpastes on color stability of a resin composite; an in vitro study

**DOI:** 10.1186/s12903-021-01956-8

**Published:** 2021-11-19

**Authors:** Sara Mehrgan, Hamid Kermanshah, Ladan Ranjbar Omrani, Elham Ahmadi, Niyousha Rafeie

**Affiliations:** 1 Dental Research Center, Dentistry Research Institute, School of Dentistry, Tehran University of Medical Sciences, Tehran, Iran; 2 Dental Research Center, Dentistry Research Institute, Department of Operative Dentistry, School of Dentistry, Tehran University of Medical Sciences, Tehran, Iran; 3 Dental Research Center, Dentistry Research Institute, Department of Operative Dentistry, School of Dentistry, Tehran University of Medical Sciences, North Kargar, 14174 Tehran, Iran

**Keywords:** Whitening toothpaste, Discoloration, Composite resin

## Abstract

**Background:**

This study aimed to compare the effects of charcoal-containing, hydrogen peroxide-containing, and abrasive whitening toothpastes on color stability of a resin composite.

**Methods:**

Forty-five specimens were fabricated of spectrum TPH3 composite resin and stored in artificial saliva for 24 h. Baseline color assessment was performed using a spectrophotometer device. Then, the specimens were randomly assigned into 5 experimental groups, namely distilled water (GC), Bencer (GB), colgate optic white (GO), perfect white black (GP) and colgate total whitening (GT) toothpastes. The specimens immersed in coffee solution for 10 min and brushed for 1 min with respective toothpaste and then stored in artificial saliva until the next day. This cycle was repeated for 30 days. After 30 days, the final color assessment was performed using the spectrophotometer. Data were analyzed using one-way ANOVA and Tukey tests.

**Results:**

Experimental groups were not significantly different in terms of Δa and ΔE values. However, ΔL and Δb values showed significant difference among the groups. Regarding Δa, GT and GC groups showed red color shift while the other groups showed green color shift. Regarding Δb, all groups showed blue color shift except GT group which showed yellow color shift.

**Conclusion:**

None of the whitening toothpastes could decrease discoloration caused by the coffee solution to the level below the perceptibility threshold except Colgate Optic White which reduced discoloration within the clinically acceptable perceptibility range.

## Background

Resin composites are commonly used in conservative dental restorative procedures due to their good esthetic properties and adequate bond to the tooth structure. Despite their satisfying aesthetic qualities, resin composite restorations are susceptible to discoloration by extrinsic factors, such as plaque accumulation and food and beverage consumption. Discoloration of these restorations might necessitate the replacement of restorations, which cost the patients, and time-consuming [1–3]. Thus, tooth whitening treatments, such as home or office bleaching and over the counter products, have been developed and used to manage discoloration [4]. It has been shown that bleaching procedures should not be performed on patients with tooth hypersensitivity, children younger than 18, and pregnant or nursing women [5]. Thus, many patients prefer to choose over the contour methods, including whitening toothpastes due to their reasonable price and easy application [6, 7]. These toothpastes are effective in tooth whitening and apply their effects by different mechanisms and agents, including chemical agents, active charcoal, and abrasive components [8]. It has been claimed that in charcoal-containing toothpastes, deposits on tooth surfaces are absorbed by activated charcoal. By brushing, charcoal and deposits stuck in charcoal irregularities, are brushed away and leave the tooth surface clean [9]. In peroxide-containing toothpastes, the peroxide agent decomposes and releases active oxygen, which reacts with the organic stains and breaks them down into non-colored organic compounds [10]. However, peroxides in toothpastes formulation are less common and challenging because of the formulation aspects [11]. Although different components present in whitening toothpastes, their whitening potentials are primarily applied by their abrasive features [6, 7]. According to the literature, whitening toothpastes effectively remove extrinsic stains. Still, they might act as a double-edged sword and make composite restorations more susceptible to discoloration by increasing the surface roughness and changing the restoration's contour. Thus, the ultimate effect of these toothpastes on the color stability of resin composites is matter of concern.

Some previous studies have investigated the effect of whitening products on the color stability of natural teeth [3, 12]. In a systematic review conducted by Soeteman et al. [13], the authors concluded that using whitening toothpastes has significantly reduced the surface staining of natural teeth compared to conventional toothpastes. Still, the information on the impact of these products on the color stability of resin composites is limited in the literature. According to Demir et al. [14] and Manis et al. [12], whitening toothpastes decreased the composite discoloration after immersion in wine and coffee, respectively. However, no toothpaste could decrease ΔE below the clinical acceptable level.

It has been shown that coffee is consumed by a large population and has a significant potential for staining both teeth and composite restorations due to its high temperature [15] and acidity [16]. Thus, the effect of whitening toothpastes on the discoloration caused by coffee consumption is a matter of concern and requires further evaluation.

The present in vitro study aimed to investigate the effects of four whitening toothpastes with different mechanisms of action, including a toothpaste with high abrasive potential (Colgate Total Whitening), two toothpastes containing active charcoal (Bencer charcoal and Perfect White Black), and a toothpaste containing hydrogen peroxide (Colgate Optic White) on the color stability of a resin composite.

## Methods

### Sample preparation

In the present study, 45 composite samples (Spectrum TPH, Dentsply Sirona Inc., Charlotte, North Carolina, USA) were fabricated in disc form shapes by compacting composite in a stainless steel mold (2 mm of diameter and 7 mm of height). A polyester matrix and glass slab were placed on both sides of the mold to press the composite with a glass slab to smooth the composite surfaces. The samples were cured by a light cure device (Woodpecker LED Curing, Guilin Woodpecker Medical Instrument Co., Guilin, China) with 1000 mW/cm^2^ of power intensity for 20 s, from each side of the mold. A radiometer (Woodpecker LM-1 Light Meter, Guilin Woodpecker Medical Instrument Co., Guilin, China) was used to calibrate the light cure intensity periodically. Samples were polished using 1200, 2400, and 4000 grit aluminum oxide abrasive disks (Extec, Enfield, CT, USA) and then stored in artificial saliva for 24 h. The samples were divided into five groups (n = 9) randomly and brushed daily for 30 consecutive days using different toothpastes as follows:

- Control: No toothpaste, only distilled water
- GO: Colgate optic white
- GT: Colgate total whitening
- GP: Perfect white black
- GB: Bencer charcoal

Table 1 summarizes the formulation of the toothpastes and manufacturers used in the present study.

**Table 1:** Ingredients and manufacturer of toothpastes used in the present study

Toothpaste	Ingredients	Manufacturer	Groups	Major whitening mechanism
Colgate optic white	Calcium pyrophosphate, propylene glycol, PEG/PPG 116/66 copolymer, PEG-12, glycerin, PVP, flavor, sodium lauryl sulfate, sodium saccharin, phosphoric acid, sucralose, butylated hydroxytoluene, water, sodium monofluorophosphate 0.76% (0.15% w/v fluoride ion), tetrasodium pyrophosphate, silica, hydrogen peroxide	Colgate Palmolive Company, New York, NY, USA	GO	Hydrogen peroxide
Colgate total whitening	Water, glycerin, sorbitol, sodium lauryl sulfate, flavor, cellulose gum, propylene glycol, carrageenan, sodium saccharin, titanium dioxide sodium fluoride 0.24% (0.15% w/v fluoride ion)—hydrated silica, PVM/MA copolymer, sodium hydroxide, triclosan 0.30%	Colgate Palmolive Company, New York, NY, USA	GT	Abrasivity
Perfect white black	Water, sorbitol, hydrated silica, glycerin, pentasodium triphosphate, sodium lauryl sulphate, aroma, PEG-32, cellulose gum, sodium fluoride, sodium saccharin, charcoal power and limonene, tetrasodium pyrophosphate, cocamidopropyl betaine	Beverly Hills Formula Company Dublin, Ireland	GP	Active charcoal components
Bencer charcoal	Deionized water, dicalcium phosphate dihydrate, glycerin, sorbitol, thickener silica, abrasive silica, sodium lauryl sulfate, mint allowed flavor, sodium carboxy methyl cellulose, polyetylene glycol 1500, sodium mono fluoro phosphate, methyl paraben,, saccharin sodium, propyl paraben menthol, activated carbon, tetra sodium pyro phosphate	Sormeh Company, Tehran, Iran	GB	Active charcoal components

### Surface treatments; immersion in coffee solution and tooth brushing

Before daily brushing, all samples were immersed in 2 ml of coffee solution for 10 min, at room temperature and under constant agitation. The coffee solution was made by mixing coffee powder (NESCAFÉ Red Mug, Nestle Corp., Vevey, Switzerland) with boiling water according to the manufacturer's instruction and cooled to room temperature. Then, toothpaste slurry was made using toothpaste and distilled water in a 3:1 ratio by weight. Each sample was brushed with toothpaste slurry by a customized automatic brushing machine for 1 min at a speed of 120 cycles/min. After brushing, samples were stored in artificial saliva at 37 °C. The composition of artificial saliva was similar to that of Viana et al. study [17]. These procedures were repeated daily for 30 consecutive days. The automatic brushing device simulated a back and forth motion within a 5 mm range. 120 cycles of daily brushing used in this study correspond to the situation that a person brushes three times a day, each with 40 cycles. The toothbrushes (Extra clean, Colgate-Palmolive Co., New York, NY, USA) used in the machine were replaced every four days; according to the study conducted by Gundavarapu et al. [18], it has been suggested that tooth brushes should be renewed every 3–4 months which corresponds to 7200–9600 cycles of tooth brushing. The bristles' hardness of the tooth brushed used in the present was medium.

### Color assessment

The color of the samples was assessed at two times:

*At the baseline*: After sample preparation and 24 h of immersion in artificial saliva, the samples were dried, and baseline L^*^, a^*^, and b^*^ values of each sample were measured by a spectrophotometer (Easyshade, VITA Zahnfabrik Co., Badsackingen, Germany).

**Fig. 1:**
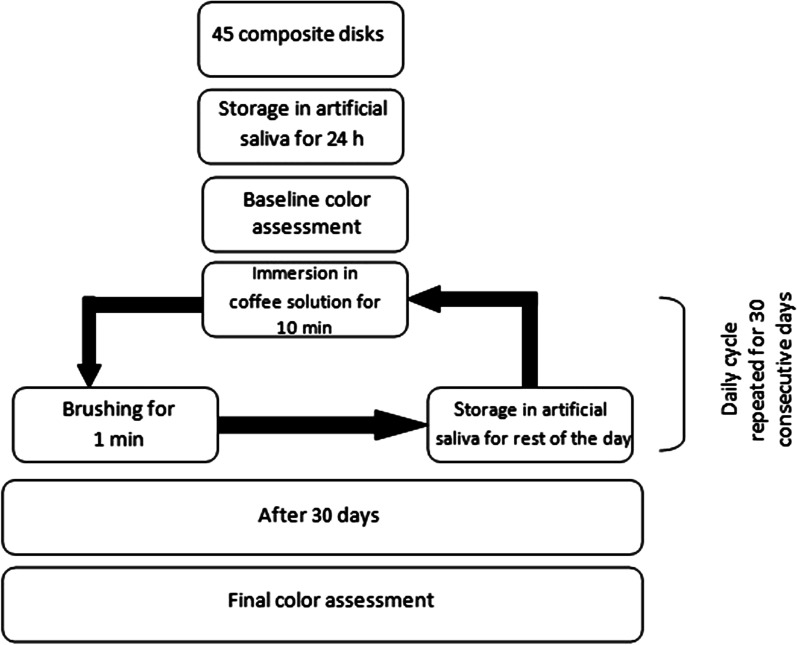
Methodology used in the present study

*After surface treatment*: After 30 days of daily tooth brushing and immersion in coffee, all samples were ultrasonically cleaned and dried. Subsequently, the spectrophotometer device assessed their colors. Figure 1 summarizes the study methodology.

Each time, the color assessment was performed using a special jig fabricated of putty (Express STD Putty, 3M-ESPE, Minnesota, USA) to ensure the assessment's reproducibility. The overall color change of samples (ΔE) was calculated using the formula below:

$$\begin{aligned} & \Delta {\text{E}} = \left[ {\left( {\Delta {\text{L}}^{*} } \right)^{{2}} + \left( {\Delta {\text{a}}^{*} } \right)^{{2}} + \left( {\Delta {\text{b}}^{*} } \right)^{{2}} } \right]^{{{1}/{2}}} \\ & \Delta {\text{L}} = {\text{L}}_{{{\text{after}}\,{\text{treatment}}}} {-}{\text{L}}_{{{\text{baseline}}}} \\ & \Delta {\text{a}} = {\text{a}}_{{{\text{after}}\,{\text{treatment}}}} {-}{\text{a}}_{{{\text{baseline}}}} \\ & \Delta {\text{b}} = {\text{b}}_{{{\text{after}}\,{\text{treatment}}}} {-}{\text{b}}_{{{\text{baseline}}}} \\ \end{aligned}$$

### Statistical analysis

Data analysis was performed using one-way ANOVA and Tukey test (for pairwise comparisons) in SPSS software (SPSS Inc., Chicago, IL, USA). The significance difference level was considered less than 0.05 (P value < 0.05).

## Results

The means and standard deviations of Δa, Δb, ΔL, and ΔE values are presented in Table 2. According to ANOVA results, five experimental groups were not significantly different in terms of Δa and ΔE values (P value = 0.19 and P value = 0.28, respectively). However, ΔL and Δb values showed significant differences among the groups (P value = 0.004 and P value = 0.05, respectively). Tukey test was performed for pairwise comparison among the groups for ΔL and Δb values. According to the results of Tukey test, a significant difference was found between GC and GT (P value = 0.007), GB and GT (P value = 0.02), and GP and GT groups (P value = 0.02) in terms of ΔL. For Δb, a significant difference was presented between GP and GT (P value = 0.04).

**Table 2:** Mean and Standard deviation of Δa, Δb, ΔL, and ΔE parameters in different experimental groups

	Control	GB	GO	GT	GP
Δa	− 0/01 ± 0/58^a^	− 0/04 ± 0/28^a^	− 0/23 ± 0/37^a^	0/15 ± 0/29^a^	− 0/24 ± 0/33^a^
Δb	− 0/56 ± 1/76^ab^	− 1/39 ± 2/33^ab^	− 0/49 ± 1/91^ab^	1/1 ± 2/48^a^	− 1/77 ± 1/62^b^
ΔL	− 4/16 ± 2/02^a^	− 3/74 ± 2/67 ^a^	− 2/09 ± 2/19^ab^	− 0/1 ± 3/18^b^	− 3/8 ± 1/43^a^
ΔE	4/56 ± 2/01^b^	4/74 ± 2/3^b^	2/9 ± 2/09^b^	3/52 ± 1/95^b^	4/37 ± 1/78^b^

Control; distilled water, GB: Bencer, GO: Optic White, GT: Total White, and GP: Perfect White. Different lowercase letters indicate significant differences (p < 0.05). The same lowercase letter indicates lack of statistically significant difference between the two subgroups (p > 0.05)

In terms of Δa, GC and GT groups showed a shift toward redness, and other groups showed a shift toward greenness. Regarding Δb, all groups showed a shift toward blueness, except GT group, which showed a shift toward yellowness.

## Discussion

The present study investigated the effects of four kinds of whitening toothpastes with different mechanisms of action, including an abrasive toothpaste (Colgate total whitening), two active charcoal containing toothpaste (Bencer charcoal and Perfect White Black), and a hydrogen peroxide-containing toothpaste (Colgate Optic White) on the color stability of resin composites.

In the present study, we evaluated Δa, Δb, ΔL, and ΔE since many studies have reported that Δb and ΔE parameters have changed dramatically after whitening treatments [19]. The shift in Δb parameter, from yellowness to blueness, is attributed to a whiter color [20, 21].

The spectrophotometer can detect ΔE values even less than 1.5 while the human eye cannot perceive ΔE values less than 3.3. Previous studies have considered different ΔE values as an acceptable threshold in clinical settings [22, 23]. We considered ΔE = 3.3 as a perceptibility threshold in the present study.

A conventional hybrid composite (Spectrum TPH) was used for this study since its physical properties including diametral tensile strength, compressive strength, flexural strength, and depth of cure are superior or comparable with those of microfill and packable resin composites which make it a reliable and popular choice for clinical application [24].

The coffee solution was used in the staining procedure since coffee is consumed by a large population and has a significant potential for staining both teeth and restorations. Besides, coffee causes resin composite discoloration due to its high temperature [15] and acidity [16]. Moreover, in addition to surface staining, coffee also causes subsurface staining because its polar and delayed-release stains are absorbed by the composite surface [25, 26].

The results of the present study revealed that there was no significant difference among the experimental groups for ΔE and Δa parameters; however, a significant difference was noted among the groups regarding Δb and ΔL parameter.

Our results were inconsistent with those of Bezgin et al. [22]. According to their results, tooth brushing using conventional toothpastes decreased the color change of the samples after 60 consecutive days; all samples showed ΔE less than 3.3. In our study, however, ΔE less than 3.3 was only presented by the GO group. In Bezgin et al. study, Coca, chocolate milk, and juice were used for staining the samples while we used the coffee solutions in the staining procedure. This may account for our different results since coffee causes more prominent discoloration than the beverages Bezgin et al. had used in their study [16].

Furthermore, the toothpastes used in their study were conventional in contrast to the present study, in which whitening toothpastes were used.

Demir et al.[14] Investigated the effect of whitening toothpaste with various mechanisms of action on the color stability of a resin composite following immersion in red wine; according to their results only brushing with Colgate Optic White toothpaste significantly decreased the discoloration caused by wine. These findings are consistent with our results which showed Colgate Optic White could decrease ΔE within the clinical acceptability range (ΔE = 2.9).

On the other hand, Manis et al. [12] concluded that none of the whitening toothpastes used in their study could decrease ΔE within the clinical acceptability range as opposed to the results of the present study. The possible explanation might be due to the different resin composite compositions including particle size and resin matrix composition used in the two studies.

It is worth mentioning that different methodology including different types of composite, toothpastes, the number of brushing cycles, and staining procedure used in the present study, makes it difficult to compare our results with those of other studies.

With regard to ΔE, the lowest value was noted in GO group, followed by GT group, GP group, Control group, and GB group. However, ΔE was not significantly different among these groups.

GO group was the only group with ΔE within the clinical acceptability range (ΔE = 2.9). Colgate Optic white toothpaste contains both chemical (hydrogen peroxide) and abrasive (silica, calcium, and pyrophosphate) agents in its formulation. These abrasive and chemical agents' synergic effect has contributed to more effective removal of surface and subsurface stains caused by coffee. Moreover, peroxide components have probably oxidized the subsurface stains and altered their absorption spectrum in such a way that human eyes could not perceive their color.

The second lowest ΔE was noted in the GT group. Colgate Total whitening toothpaste contains TiO_2_ pigments. It is possible that the precipitation of TiO_2_ pigments on the composite surface have covered the yellow stains caused by the coffee solution and have decreased ΔE.

The Control group showed the highest ΔE after the GB group. According to the studies, the high number of tooth brushing cycles leads to degradation of the composite resin, increasing surface roughness, and decreasing surface brightness [27]. Similarly, the tooth brushing cycles might have increased the surface roughness of composite resin in the present study and made it more susceptible to discoloration, but this discoloration has been improved relatively in GO, GT, and GP groups due to the whitening effects of toothpastes. On the other hand, the control group samples were only brushed by distilled water, and the lack of polishing and whitening effects of toothpastes may account for higher ΔE values in this group.

Finally, the highest ΔE was noted in the GB group. Bencer toothpaste contains active charcoal. The efficacy of charcoal-containing toothpastes depends on several factors, including the size, form, and abrasiveness of charcoal particles [28]. Since there was not adequate information about these factors in Bencer toothpaste, we could not explain the exact mechanism which had resulted in higher ΔE values in this group.

Regarding Δb, a significant difference was found between GT and GP groups which showed a shift toward yellowness and blueness, respectively. The GT group showed the highest shift toward yellowness that might be related to the high relative dentin abrasivity (RDA) value of Colgate Total Whitening, which caused a lot of abrasion on the composite surface. As a result, coffee stains were absorbed by the rough surface and caused subsurface staining.

The GP showed a shift toward blueness. It is speculated that the carbon in Perfect White Black toothpaste has absorbed coffee stains and resulted in this shift. The shift toward blueness also occurred in other carbon-containing toothpaste (Bencer); however, its Δb was not significantly different from the other groups.

The most important limitation of the present study is that the laboratory setting used in this study cannot completely simulate the oral environment; in the oral cavity, teeth, restorations, and soft tissues are constantly cleaned by circulating saliva and their exposure time to materials and stains decreases significantly. Besides, the artificial saliva used for storing the samples lacks the enzymes and pellicles presented in saliva. Thus, generalization of the results obtained from this study to clinical settings must be made cautiously. It should be noted that many coffee consumers are cigarette smokers; we did not evaluate the effect of smoking on the color change of the samples in our study.

## Conclusions

Within the limitations of the present study, the results revealed after 30 consecutive days, none of the whitening toothpastes could decrease discoloration caused by the coffee solution to the level below the perceptibility threshold except Colgate Optic White. Thus, the use of Colgate Optic White might be beneficial for coffee drinkers who experience discoloration of their composite discoloration. However, further clinical studies are required to confirm these results.

## Data Availability

The datasets used and/or analysed during the current study are available from the corresponding author on reasonable request.

## Abbreviations

GO: Colgate optic white toothpaste
GT: Colgate total whitening toothpaste
GP: Perfect white black tooth paste
GB: Bencer charcoal tooth paste
